# Socioecological model-based design and implementation principles of lower limb preservation programs as partners for limb-loss rehabilitation programs— A mini-review

**DOI:** 10.3389/fresc.2022.983432

**Published:** 2022-12-12

**Authors:** Vipul Khetarpaul, John P. Kirby, Patrick Geraghty, John Felder, Prateek Grover

**Affiliations:** Washington University School of Medicine in St. Louis, St. Louis, MO, United States

**Keywords:** amputation, lower limb, program development, rehabilitation, reconstructive surgery, socioecological model of health, vascular surgical procedure, wound healing

## Abstract

People with lower limb loss, especially of dysvascular etiology, are at substantial risk for both ipsilateral and contralateral reamputation. Additionally, while not as well documented for reamputation, there is recognition that amputation incidence is influenced by not only sociodemographic factors such as sex, race, socioeconomic status, but also by system factors such as service access. A systems strategy to address this disparity within the field of limb-loss rehabilitation is for Limb-loss Rehabilitation Programs (LRP) to partner with medical specialists, mental health professionals, and Limb Preservation Programs (LPP) to provide comprehensive limb care. While LPPs exist around the nation, design principles for such programs and their partnership role with LRPs are not well established. Using a socioecological model to incorporate hierarchical stakeholder perspectives inherent in the multidisciplinary field of limb care, this review synthesizes the latest evidence to focus on LPP design and implementation principles that can help policymakers, healthcare organizations and limb-loss rehabilitation and limb-preservation professionals to develop, implement, and sustain robust LPP programs in partnership with LRPs.

## Introduction

50,000–60,000 major amputations occur every year, with peripheral arterial disease (PAD) and diabetes mellitus being the most common causes (1). Rates of reamputation remain high following major amputations, with 1-year and 5-year contralateral major amputation rates being 5.7% and 11.5%, respectively. Even after minor amputations, 1-year and 5-year rates of are high, being 3.2% and 8.4% for contralateral major amputations, and 10.5% and 14.2% for ipsilateral major amputations, respectively. Risk of contralateral amputation increases with renal disease and atherosclerosis with or without diabetic neuropathy (2), with ipsilateral reamputation rates in diabetic patients being 5, 12, and 13% at 1-year, 3-year, and 5-year periods (3). 5-year mortality rates range from 29% to 69% following minor amputations, and from 52% to 80% for major amputations (4). This high risk of reamputation following limb loss, especially with dysvascular etiology, requires comprehensive limb care with not only Limb-Loss Rehabilitation Programs (LRP) to manage the complex care and comorbidities of the post limb-loss patient population, but also partnership with Limb-Preservation Programs (LPP) to minimize subsequent limb-loss. This mini-review article focuses specifically on LPPs as partners for LRPs to enable comprehensive limb care.

The overarching vision of LPPs is attainment of pain-free, functioning limbs that enable continued independence with excellent quality of life, while using limited resources optimally to maximize care delivery through coordinated multidisciplinary team care. While most robust LPP programs exist at tertiary-level academic centers (5) and within the Veterans Healthcare system (6), many of the design principles and strategies for developing and implementing such highly coordinated LPPs can be adapted by other healthcare organizations to improve overall limb-care health equity across geographic boundaries.

The authors utilize an established public health concept, the socio-ecological model (7), as a framework to present a structured hierarchical perspectives-inclusive overview of LPP design principles and implementation strategies. The article is comprised of 3 sections, describing the need for LPP services (Section “Need for limb-preservation program (LPP) services”), socioecological-level based limb care model structure focusing on partnerships between LPP and other programs (Section “Socio-ecological level-based limb care structural model”), and multilevel implementation strategies that can be adopted by healthcare organizations for LPP development (Section “Multilevel interventions and implementation strategies for LPP development”).

## Need for limb-preservation program (LPP) services

The goal of LPPs is early recognition of disease to facilitate preventive care and timely intervention, with individualized plans and ultimate reduction in amputation risk and rates. LPPs vary in terms of structure, and can include a combination of vascular, and limb reconstruction services.

### Wound care services

Nonhealing wounds affect 3 to 6 million people in US with annual healthcare costs exceeding $3 billion (8). Studies have reported the occurance of foot ulcerations in as many as 85% patients needing amputation (9). Hence, dedicated wound care teams are needed to optimize clinical outcomes and cost (10).

### Vascular services

Trans-Atlantic Inter-Society Consensus Working Group II estimates PAD-related major amputation incidence of 12–50/100,000 (11). Threefold-increase in endovascular interventions was shown to decrease amputation rates by 25% despite the increasing prevalence of diabetes in Medicare beneficiaries from 1996 to 2006 (12). However, this may not be enough, with only 68.4% undergoing arterial testing prior to amputation in 17,463 Medicare non-traumatic amputees from 2000 to 2010. Although some clinical scenarios (such as fulminant infection) preclude attempts at limb salvage, diagnosis, and treatment of limb ischemia prior to undertaking major amputation remains a key goal (13). Such decision-making and interventions require vascular services.

### Limb reconstruction services

Residual limb issues that limit optimal prosthetic device fitting and use, such as chronic wounds, prominent bone edges, redundant soft tissue, and neuromas (14) are some indications for review and further management with limb reconstruction services offered by specialties such as Plastic surgery and Orthopedic Surgery.

Ideally, these three services should coexist and collaborate to provide comprehensive limb preservation care, in partnership with rehabilitation, primary care, and mental health services. Depending upon resources, specialists in infectious diseases, endocrinology, cardiology, interventional radiology, plastic surgery, and pain management should be included in the program. Mental health resources can include psychologist, counselor, or psychiatrist services. Rehabilitation services provided by Physical Medicine and Rehabilitation (PM&R), occupational and physical therapy specialists should be offered to maintain a high level of functioning.

Healthcare institutions and organizations seeking to develop, expand or refine their ability to provide comprehensive limb care would benefit from using a conceptual model to comprehensively understand their current structure (Section “Socio-ecological level-based limb care structural model”), followed by determination of implementation strategies and interventions to address context-specific factors (Section “Multilevel interventions and implementation strategies for LPP development”). Both the Limb Care model structure and implementation strategies are described using the socio-ecological model to incorporate hierarchical stakeholder perspectives inherent in the multidisciplinary field of limb care.

## Socio-ecological level-based limb care structural model

**Figure 1:**
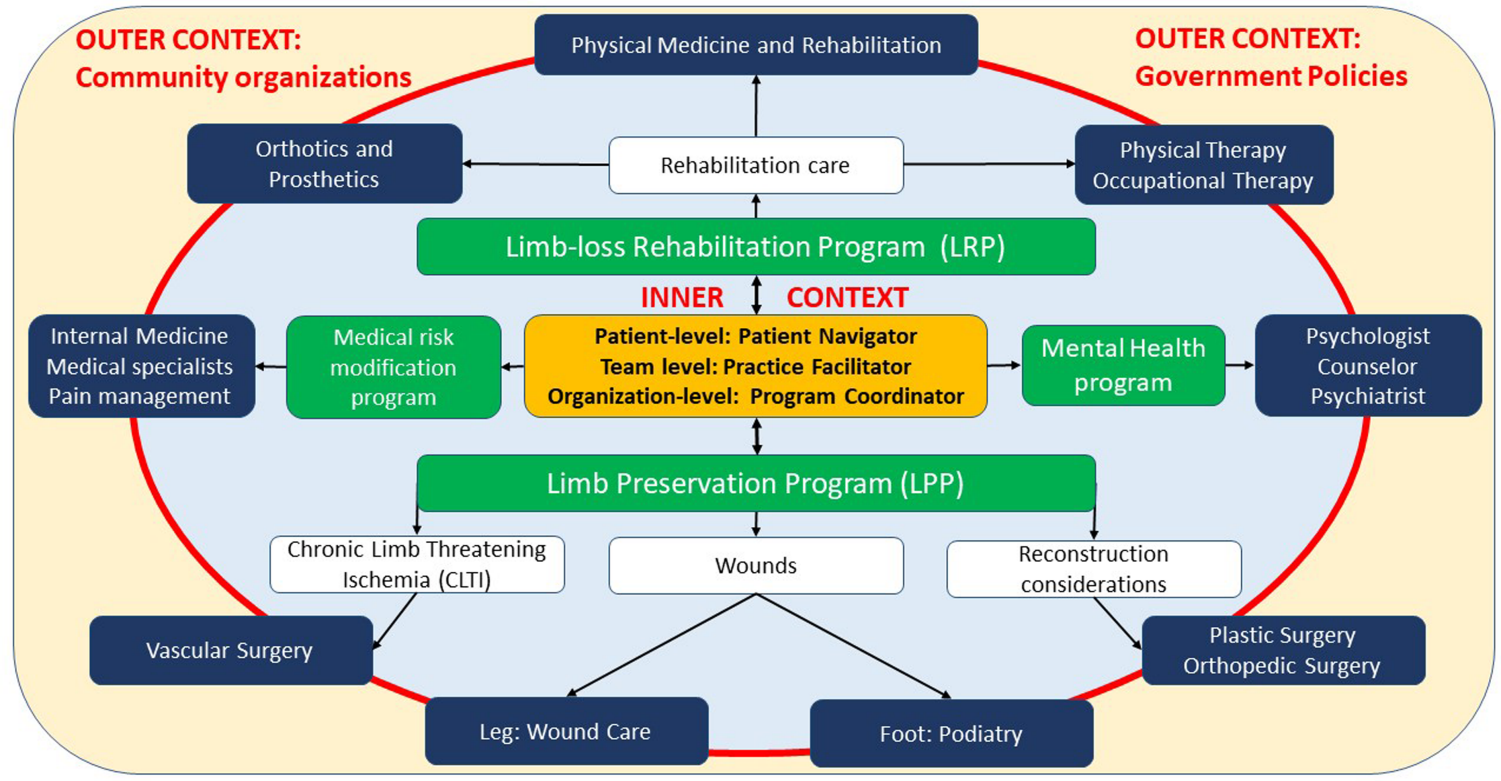
Model for comprehensive limb care though partnership between limb-loss rehabilitation, limb preservation, medical risk modification, and mental health programs.

LPPs to address diabetic foot ulcers (15) and ischemic limbs (16) provide valuable insight into program design. To further expand on how LPPs can integrate with other services to provide comprehensive limb care, the authors present a Limb Care model that illustrates LPP partnerships with LRPs and other synergistic programs (Figure 1).

The model structure includes four main constructs: context (outer yellow box and inner light blue oval, separated by a red boundary), programs (green boxes), services (dark blue boxes), and central coordination (orange box). Outer Context and Inner Context are implementation science model [e.g., EPIS (17)] constructs that reflect the ability of a program to influence factors at various socio-ecological levels. ***Outer Context*** includes *system and community socio-ecological levels*. Factors at these levels are entrenched and not easily influenced by a program. Implementation strategies at these levels are described in Sections “System level: understand policies and payment mechanisms” and “Community level: utilize patient resources and evidence-based guidelines”. ***Inner Context*** include *patient, provider, and organization socio-ecological levels.* Factors at these levels are often within a programs' sphere of influence. Implementation strategies at these levels are described in Sections “Organization level: develop processes for quality, coordination, and program sustainability”, “Provider-level principles: stay up to date with standard-of-care diagnosis and management practices” and “Patient-level principles: empower through education and navigation strategies”.

The partnership of LPPs with other programs lies within Inner Context. **LPP** includes four services, that are accessed based upon the specific indication for limb preservation. Vascular surgery services are utilized for limb threatening ischemia. Wound care and podiatry services are utilized based upon wound location proximal and distal to the ankle, respectively. Plastic and Orthopedic Surgery services are utilized to address reconstruction needs, as described in Section “Need for limb-preservation program (LPP) services”. Three other programs are included within inner context. **LRPs** include PM&R, orthotics and prosthetics and therapy services to address rehabilitation needs. **Medical Risk Modification Programs** include internal medicine, pain management, and other medical specialists to minimize medical risk for reamputation. **Mental Health Programs** can include psychologist, counselor, and psychiatry services to optimize psychological health.

***Central coordination*** is at the core of this model's LPPs partnerships with the other programs. Interventions for successful coordination have been described for healthcare programs such as maternal health, behavioral health, and oncology. Examples of some of these interventions that are potentially applicable for limb care program coordination are listed in the Central coordination box, and include program coordinators (18) (*organization-level*), practice facilitators (19) (*provider-level*), and patient navigators (20) (*patient-level*).

**Figure 2:**
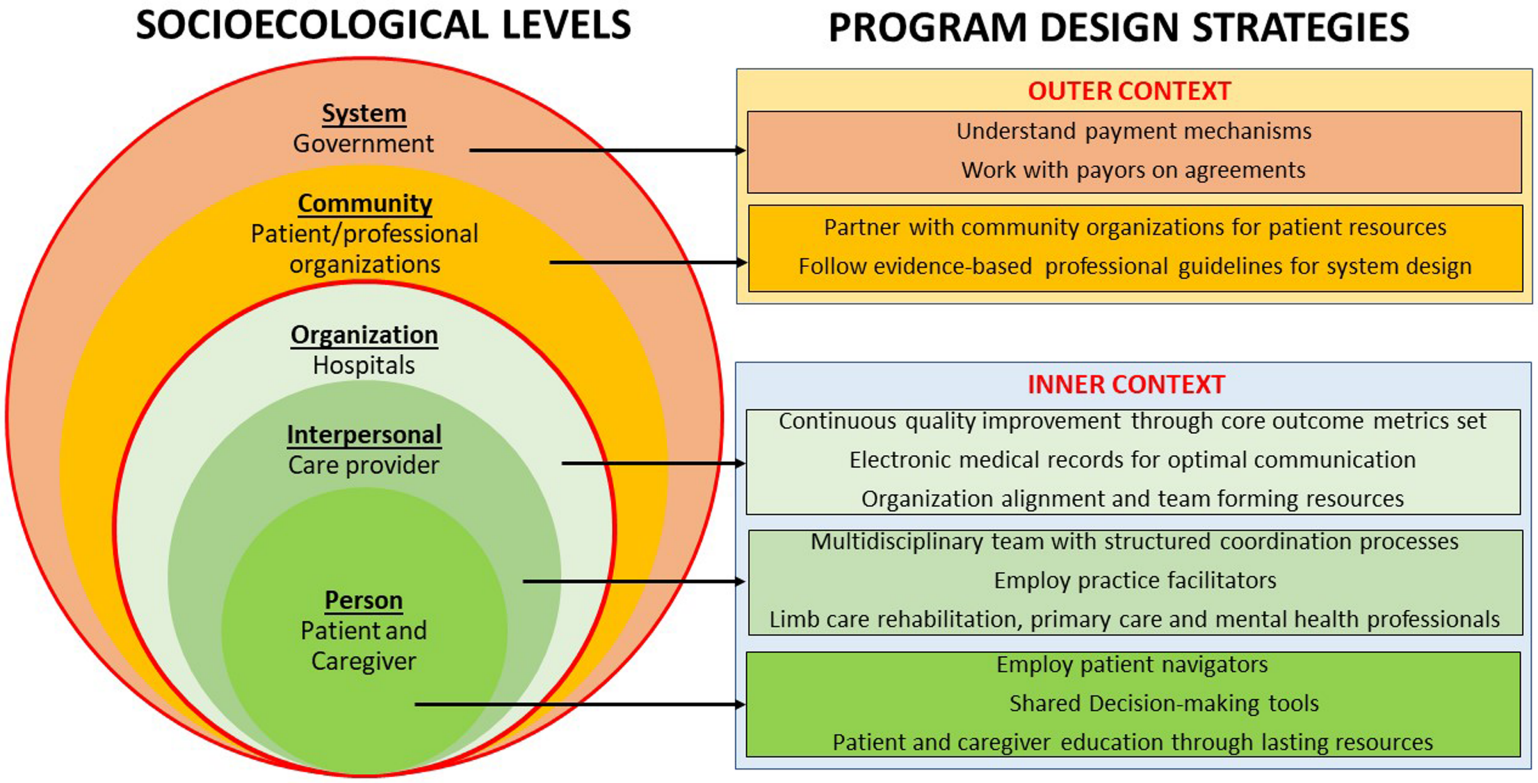
Socioecological level-based limb preservation program (LPP) design strategies for inner and outer context.

Once healthcare organizations have a good understanding of their limb care program structure and resources, determination of multilevel interventions and implementation strategies for LPP development and refinement is the next step. Examples of such interventions and implementation strategies corresponding to socio-ecological levels are presented in Section “Multilevel interventions and implementation strategies for LPP development” (Figure 2).

## Multilevel interventions and implementation strategies for LPP development

### System-level principles: understand policies and payment mechanisms

National health policies and payment mechanisms influence access for people seeking limb-preservation services. An example is higher amputation rates for people without insurance and with Medicaid (21). These payment mechanisms also influence provider payment and organizational reimbursement. Programs must understand their system factors and work with payors to maximize their ability to provide care.

### Community-level principles: utilize patient resources and evidence-based guidelines

#### Patient's physical and social environment

Patient's physical and social environment (22) can limit patient access to healthcare. Appointment adherence can be addressed with interventions such as transport and appointment reminders. Home wheelchair accessibility limitations can be addressed by home assessment and modifications, which may require partnerships with community rehabilitation agencies such as vocational rehabilitation. Community mobility can be enabled by power wheelchairs/scooters. Prescription must be customized to individuals based upon potential for overuse injuries balanced with deconditioning.

#### Patient advocacy organizations

Patient advocacy organizations such as Amputee Coalition (https://www.amputee-coalition.org/) and Limb Preservation Foundation (https://limbpreservation.org/) offer resources for persons with threatened limb loss such as peer mentors as well as educational resources in online and print formats, such as the First Step Manual in English (https://shop.amputee-coalition.org/first-step-p42.aspx) and Spanish (https://shop.amputee-coalition.org/first-step-en-espaol-una-gua-para-adaptarse-a-la-prdida-de-extremidades-p26.aspx). Programs should make use of these community resources for patient education.

#### Professional organizations

Professional organizations are increasingly collaborating for standardization of care and quality guidelines. Evidence-based wound care guidelines by World Healing Foundation have enabled care for not only common etiologies, but also uncommon etiologies such as rheumatologic disorders with vasculitis, and dermatologic abnormalities with pyoderma (23, 24). Peripheral Academic Research Consortium has provided consensus definitions for standardization of clinical studies with Peripheral Arterial Disease/Chronic Limb Threatening Ischemia, with development of evidence-based clinical practice guidelines (25). Global Vascular Guidelines jointly released by the Society for Vascular Surgery, European Society for Vascular Surgery, and World Federation of Vascular Societies (26) incorporate wound, ischemia, and foot infection (WiFi) staging (27) and Global Anatomic Staging System (GLASS) methodology (28) for anatomic assessment and triaging into medical management and surgical intervention groups. The LEAP program developed in 1992 by HRSA (https://www.hrsa.gov/hansens-disease/leap) is a good example of wound prevention principles for people with impaired foot sensation that can be implemented by primary care. Programs should embed standard guidelines within their care programs.

### Organization-level principles: develop processes for quality, coordination, and program sustainability

Programs should define outcome metric sets for continuous quality improvement. Examples of clinical metrics include wound healing rates, healing time exceeding 30 or 60 days, wound care/vascular technique type, and amputation rate. Example of utilization metrics include charges billed to insurance company, cost incurred to patient, and collection by the facility. Examples of functioning and rehabilitation metrics include pain control (impairment), distance and speed of ambulation (activity) and return to work (participation). Patient reported outcome measures (PROMs) including satisfaction with care are useful to understand patient perspective.

Programs should employ electronic medical records (EMR) for optimal information sharing and care coordination. Given the heterogeneity in patient characteristics, wound-care diagnostic protocols and therapies (29, 30), scientific evidence for using EMR data for a precision medicine approach, matching patients with best therapies for best clinical outcomes and resource utilization is a work in progress.

While effective multi-disciplinary teams are effective in decreasing amputation prevalence (31), dedicated support from the organizations that invest in education and team-forming resources is vital. The role of organizational champions cannot be overstated (32). Practice facilitators may play an integral role in team-forming as well.

### Provider-level principles: stay up to date with standard-of-care diagnosis and management practices

#### Diagnostic approach

A comprehensive history should elucidate information on potential risk factors for wounds and vascular disease such as injury, diabetes, neuropathy, ill-fitting shoes, smoking, and sedentary lifestyle. Physical exams should include a detailed evaluation for changes related to vascular insufficiency, neuropathy, and foot architecture. Diagnostic testing should be guided by the history and exam as well as resources available. This can range from simple screens such as Semmes-Weinstein monofilament exam, to digital photography and vascular lab studies, to even more sophisticated tests such as tissue concentration of oxygen, and near infra-red photography (33). Comprehensive holistic evaluation should be accompanied by realistic expectation setting (34, 35).

#### Wound care interventions

Fifty percent reduction in wound area by 30 days (36) is a clinically accepted goal. Delay in wound healing should prompt evaluation for contributory factors and consideration for more advanced diagnostic and management techniques. Early debridement can be both diagnostic and therapeutic. Chronic ulcerations often need serial debridement and biopsies, and abnormal bony architecture may need correction, combined with off-loading positioning education, mattress overlays, shoes, and sometimes total contact casting (37). Inflammatory, nutritional, or collagen-based deficiencies hampering wound healing should be addressed as well. Once a clean wound bed is obtained, further support can be provided by re-epithelialization techniques from grafting to cultured skin grafts and other biological implants, to skin and tissue scaffolds. Advanced modalities include hyperbaric oxygen to stimulate neo-angiogenesis (38, 39), e-stimulation and ultrasound (40), complex angiosomal reconstruction and negative pressure either alone or in combinations with skin grafting options, pro-healing scaffolds, biologically stimulating dressings, and pluri-potent cell lines. Classification of wounds, appropriate diagnosis and intervention strategy selection should be based upon the latest clinical practice guidelines (41), available resources, and shared decision making with patients and their caregivers.

#### Vascular care interventions

Interventions can range from vein bypasses and open aortobifemoral reconstructions to endovascular interventions such as drug delivery to limit restenosis, lithotripsy of calcified lesions, pedal loop interventions, and deep venous arterialization for desert foot. Hybrid interventions that combine open and endovascular options responsive to individual factors such as anatomy, disease burden and co-morbidities have been steadily increasing [6.1% in 2010 to 32% in 2017 (42)]. Efforts should focus on the restoration of inline flow to the wound-specific angiosome, which results in the best chance of wound healing. Surgical intervention recommendation for claudicants depend upon anatomic location, with better chances of symptom improvement with aortoiliac and common femoral segment who fail medical treatment alone. Interestingly, vascular interventions in claudicants have not shown a reduction in progression to limb-threatening ischemia and amputation (43). Regenerative medicine approaches like gene and cell therapy for CLTI should be restricted to randomized controlled trials.

#### Reconstructive interventions

Contemporary thought emphasizes that amputation surgery itself be considered a reconstructive surgery (44). Reconstruction of a functional residual limb is the goal, and this should include surgical measures to prevent chronic pain and biomechanical or soft tissue deformities that make ambulation with a prosthesis difficult. Residual limb issues that limit prosthetic device fitting and use, such as chronic wounds, neuromas, prominent anatomy, and redundant tissue are indications for review and further management with limb reconstruction services offered by specialties such as Plastic surgery and Orthopedic Surgery. Recent innovations in peripheral nerve surgery such as targeted muscle reinnervation (TMR) (45–47) and regenerative peripheral nerve interface (RPNI) (48) techniques have improved our ability to surgically treat painful neuromas and phantom pain. Osseointegration interfaces for prosthetics are now FDA approved for transfemoral amputation and increasingly available. For appropriate patients, they offer the advantages of improved skeletal alignment with the prosthesis, subsequent reduction in energy expenditure, simplified donning and doffing, and improvement in quality of life (49). Adoption may be slow related to surgical expertise as well insurance issues.

#### Medical risk factor modification

Programs should address risk factor modification and medical optimization for reducing long-term risks such as cardiovascular mortality and morbidity, by managing conditions such as diabetes, chronic kidney disease and hypertension, and smoking cessation. Antiplatelet therapy with aspirin or clopidogrel reduces the risk of myocardial infarction, stroke, and death in these patients (50). High potency statin therapy reduces risk of cardiovascular mortality and amputation risk in PAD patients (51, 52). Aspirin with low dose rivaroxaban has major cardiovascular benefit and reduced need for revascularization compared with revascularization alone per the VOYAGER PAD trial (53).

#### Mental health

Both prior mental health concerns and new concerns that arise with major surgeries such as amputations (54) should be addressed by including mental health professionals such as psychologists, counselors, and psychiatrists in the care provider team. Peer-mentors offer a valuable resource for mental health as well.

#### Rehabilitation

Programs should include PM&R guided therapy, medical equipment, assistive devices (e.g., wheelchair, walker, cane) and orthotic determination, presurgical planning, postsurgical rehabilitation care, and care coordination with community resources such as peer support, vocational, recreational, and driving rehabilitation. The value to prehabilitation for improving postoperative outcome such as mobility is a promising area of study (55).

### Patient-level principles: empower through education and navigation strategies

Program should include patient education strategies, with the goal of maximizing patient understanding, participation in decision making and realistic goal setting (56). Patient navigation resources help patients to navigate complicated care-delivery systems. Examples of outcomes that can be measured include self -foot exams, adherence to medical and rehabilitation management recommendations, appointment no-shows, and quality of life metrics.

Surgical decision making should be guided by patient's expectations and health conditions.

Inability to adhere to recommendations for medical (e.g., oncologic) and other reasons may be better served with earlier tissue resection and closures rather than prolonged attempts at healing. This is because the latter can lead to potential secondary complications such as immobility-related cardio-respiratory functional decline, and long-term antibiotics-related worsening renal function. Where wounds represent a terminal condition such as those seen in cancer or Kennedy terminal ulcerations, palliative or custodial wound care may be presented as option.

## Summary

Limb care is an inter-disciplinary field. Comprehensive programs should be partnerships between LRP and LPP that address not only limb health, but also medical risk factors, mental health, and functioning, with the goal of optimizing long-term outcomes and quality of life. A multilevel approach that includes system, community, organization, provider, and patient perspectives should guide comprehensive LPP design. Major strategies should include realistic goal setting and adherence promotion with patient empowerment, multidisciplinary team-based approach that incorporates standard-of-care practices, focus on process improvement and EMRs for efficient communication, utilization of evidence-based guidelines and established patient resources, and working with payors to maximize impact.
